# Supplementary material for: Getting More Out of Biomedical Documents with GATE's Full Lifecycle Open Source Text Analytics
Source: PLoS Comput Biol. 2013 Feb 7;9(2):e1002854. doi: 10.1371/journal.pcbi.1002854 (PMC3567135; doi:10.1371/journal.pcbi.1002854)
Supplement: Dataset S1 — GATE software. Dataset S1 bundled with this paper contains a distribution of GATE (or see http://gate.ac.uk/download/). (TGZ) [file pcbi.1002854.s001.tgz › plos-gate/src/gate/jape/parser/ParseCpslPlain.html]

BNF for ParseCpsl.jj


# BNF for ParseCpsl.jj

## TOKENS

```
///////////////////
// lexical analysis
///////////////////

////////////////
// utility stuff
```

```
<DEFAULT> TOKEN : {
<#space: "\n" | "\r" | "\t" | "\f" | " ">
}
```

```
<DEFAULT> TOKEN : {
<#spaces: ("\n" | "\r" | "\t" | "\f" | " ")+>
}
```

```
<DEFAULT> TOKEN : {
<#newline: "\n" | "\r" | "\n\r" | "\r\n">
}
```

```
<DEFAULT> TOKEN : {
<#digits: (["0"-"9"])+>
}
```

```
<DEFAULT> TOKEN : {
<#letter: ["A"-"Z","a"-"z"]>
}
```

```
<DEFAULT> TOKEN : {
<#letterOrUnderscore: ["A"-"Z","a"-"z","_"]>
}
```

```
<DEFAULT> TOKEN : {
<#letters: (["A"-"Z","a"-"z"])+>
}
```

```
<DEFAULT> TOKEN : {
<#lettersAndDigits: (["A"-"Z","a"-"z","0"-"9"])+>
}
```

```
<DEFAULT> TOKEN : {
<#letterOrDigitOrDash: ["A"-"Z","a"-"z","0"-"9","-","_"]>
}
```

```
<DEFAULT> TOKEN : {
<#lettersAndDigitsAndDashes: (["A"-"Z","a"-"z","0"-"9","-","_"])+>
}
```

```
////////////////
// parsed tokens
```

```
<DEFAULT> TOKEN [IGNORE_CASE] : {
<multiphase: "Multiphase:">
}
```

```
// phases has its own lexical state so we can deal with relative paths
// pointing to grammar files
```

```
<DEFAULT> TOKEN [IGNORE_CASE] : {
<phases: "Phases:"> : IN_PHASES
}
```

```
<IN_PHASES> TOKEN : {
<path: (["A"-"Z","a"-"z","0"-"9","-","_","/","\\","."])+>
}
```

```
<IN_PHASES> SPECIAL : {
<phasesWhiteSpace: (<space>)+>
}
```

```
<IN_PHASES> SPECIAL : {
<phasesSingleLineCStyleComment: "//" (~["\n","\r"])* <newline>>
}
```

```
<IN_PHASES> SPECIAL : {
<phasesSingleLineCpslStyleComment: ";;" (~["\n","\r"])* <newline>>
}
```

```
<IN_PHASES> SPECIAL : {
<phasesCommentStart: "/*" | "#|"> : PHASES_WITHIN_COMMENT
}
```

```
<PHASES_WITHIN_COMMENT> MORE : {
<phasesCommentChars: ~[]>
}
```

```
<PHASES_WITHIN_COMMENT> SPECIAL : {
<phasesCommentEnd: "*/" | "|#"> : IN_PHASES
}
```

```
<DEFAULT> TOKEN [IGNORE_CASE] : {
<javaimport: "Imports:">
}
```

```
<DEFAULT> TOKEN [IGNORE_CASE] : {
<controllerstarted: "ControllerStarted:">
}
```

```
<DEFAULT> TOKEN [IGNORE_CASE] : {
<controllerfinished: "ControllerFinished:">
}
```

```
<DEFAULT> TOKEN [IGNORE_CASE] : {
<controlleraborted: "ControllerAborted:">
}
```

```
<DEFAULT> TOKEN [IGNORE_CASE] : {
<phase: "Phase:">
}
```

```
<DEFAULT> TOKEN [IGNORE_CASE] : {
<input: "Input:">
}
```

```
<DEFAULT> TOKEN [IGNORE_CASE] : {
<option: "Options:">
}
```

```
<DEFAULT> TOKEN [IGNORE_CASE] : {
<rule: "Rule:">
}
```

```
<DEFAULT> TOKEN [IGNORE_CASE] : {
<macro: "Macro:">
}
```

```
<DEFAULT> TOKEN [IGNORE_CASE] : {
<template: "Template:">
}
```

```
<DEFAULT> TOKEN [IGNORE_CASE] : {
<priority: "Priority:">
}
```

```
<DEFAULT> TOKEN : {
<pling: "!">
}
```

```
<DEFAULT> TOKEN : {
<kleeneOp: "*" | "+" | "?">
}
```

```
<DEFAULT> TOKEN : {
<attrOp: "==" | "!=" | ">" | "<" | ">=" | "<=" | "=~" | "!~" | "==~" | "!=~">
}
```

```
<DEFAULT> TOKEN : {
<metaPropOp: "@">
}
```

```
<DEFAULT> TOKEN : {
<integer: (["+","-"])? (["0"-"9"])+>
}
```

```
//starts a string
```

```
<DEFAULT> MORE : {
"\"" : IN_STRING
}
```

```
//reads the contents of the string
```

```
<IN_STRING> MORE : {
"\\n" : {
| "\\r" : {
| "\\t" : {
| "\\b" : {
| "\\f" : {
| "\\\"" : {
| "\\\'" : {
| "\\\\" : {
| <"\\u" ["0"-"9","A"-"F","a"-"f"] ["0"-"9","A"-"F","a"-"f"] ["0"-"9","A"-"F","a"-"f"] ["0"-"9","A"-"F","a"-"f"]> : {
| <~["\"","\\"]>
}
```

```
//finishes the string
```

```
<IN_STRING> TOKEN : {
<string: "\""> : DEFAULT
}
```

```
<DEFAULT> TOKEN : {
<bool: "true" | "false">
}
```

```
<DEFAULT> TOKEN : {
<ident: <letterOrUnderscore> (<letterOrDigitOrDash>)*>
}
```

```
<DEFAULT> TOKEN : {
<floatingPoint: (["+","-"])? ((["0"-"9"])+ "." (["0"-"9"])* (<exponent>)? (["f","F","d","D"])? | "." (["0"-"9"])+ (<exponent>)? (["f","F","d","D"])? | (["0"-"9"])+ <exponent> (["f","F","d","D"])? | (["0"-"9"])+ (<exponent>)? ["f","F","d","D"])>
}
```

```
<DEFAULT> TOKEN : {
<#exponent: ["e","E"] (["+","-"])? (["0"-"9"])+>
}
```

```
<DEFAULT> TOKEN : {
<colon: ":">
}
```

```
<DEFAULT> TOKEN : {
<semicolon: ";">
}
```

```
<DEFAULT> TOKEN : {
<period: ".">
}
```

```
<DEFAULT> TOKEN : {
<bar: "|">
}
```

```
<DEFAULT> TOKEN : {
<comma: ",">
}
```

```
<DEFAULT> TOKEN : {
<leftBrace: "{">
}
```

```
<DEFAULT> TOKEN : {
<rightBrace: "}">
}
```

```
<DEFAULT> TOKEN : {
<leftBracket: "(">
}
```

```
<DEFAULT> TOKEN : {
<rightBracket: ")">
}
```

```
<DEFAULT> TOKEN : {
<leftSquare: "[">
}
```

```
<DEFAULT> TOKEN : {
<rightSquare: "]">
}
```

```
<DEFAULT> TOKEN : {
<assign: "=">
}
```

```
<DEFAULT> TOKEN : {
<colonplus: ":+">
}
```

```
//  TOKEN: {  |   > }
// TOKEN: {  }
/* SPECIAL_TOKEN: { // catch all for Java block processing
  
}*/

////////////////////
// non-parsed tokens

// we make comments and spaces special tokens to support an editor
```

```
<DEFAULT> SPECIAL : {
<whiteSpace: (<space>)+>
}
```

```
<DEFAULT> SPECIAL : {
<singleLineCStyleComment: "//" (~["\n","\r"])* <newline>>
}
```

```
<DEFAULT> SPECIAL : {
<singleLineCpslStyleComment: ";;" (~["\n","\r"])* <newline>>
}
```

```
<DEFAULT> SPECIAL : {
<commentStart: "/*" | "#|"> : WITHIN_COMMENT
}
```

```
<WITHIN_COMMENT> MORE : {
<commentChars: ~[]>
}
```

```
<WITHIN_COMMENT> SPECIAL : {
<commentEnd: "*/" | "|#"> : DEFAULT
}
```

```
<DEFAULT> TOKEN : {
<other: ~[]>
}
```

## NON-TERMINALS

|  |  |  |
| --- | --- | --- |
| ``` ////////////// // the grammar ////////////// ``` |  |  |
| \_MultiPhaseTransducer | ::= | ( <multiphase> <ident> )? ( ( ( JavaImportBlock ) ( ( ControllerStartedBlock ) \| ( ControllerFinishedBlock ) \| ( ControllerAbortedBlock ) )\* ( SinglePhaseTransducer )+ ) \| ( <phases> ( <path> )+ ) ) <EOF> |
| ``` // _MultiPhaseTransducer ``` |  |  |
| SinglePhaseTransducer | ::= | <phase> <ident> ( ( <input> ( <ident> )\* ) \| ( <option> ( <ident> <assign> ( <ident> \| <bool> ) )\* ) )\* ( ( Rule ) \| MacroDef \| TemplateDef )\* |
| ``` // SinglePhaseTransducer  // if there is a block, set the javaimports to the java block specified, // otherwise set it to the default block ``` |  |  |
| JavaImportBlock | ::= | ( <javaimport> <leftBrace> ConsumeBlock )? |
| ControllerStartedBlock | ::= | ( <controllerstarted> <leftBrace> ConsumeBlock ) |
| ControllerFinishedBlock | ::= | ( <controllerfinished> <leftBrace> ConsumeBlock ) |
| ControllerAbortedBlock | ::= | ( <controlleraborted> <leftBrace> ConsumeBlock ) |
| Rule | ::= | <rule> <ident> ( <priority> <integer> )? LeftHandSide "-->" RightHandSide |
| ``` // Rule ``` |  |  |
| MacroDef | ::= | <macro> <ident> ( PatternElement \| Action ) |
| ``` // MacroDef ``` |  |  |
| TemplateDef | ::= | <template> <ident> <assign> AttrVal |
| ``` // TemplateDef ``` |  |  |
| LeftHandSide | ::= | ConstraintGroup |
| ``` // LeftHandSide   // we pass the lhs down so we can add bindings in CPEs, and the cg // so we can add PEs and create disjunctions here ``` |  |  |
| ConstraintGroup | ::= | ( PatternElement )+ ( <bar> ( PatternElement )+ )\* |
| ``` // ConstraintGroup ``` |  |  |
| PatternElement | ::= | ( <ident> \| BasicPatternElement \| ComplexPatternElement ) |
| ``` // PatternElement ``` |  |  |
| BasicPatternElement | ::= | ( ( <leftBrace> Constraint ( <comma> Constraint )\* <rightBrace> ) \| ( <string> ) ) |
| ``` // BasicPatternElement ``` |  |  |
| ComplexPatternElement | ::= | <leftBracket> ConstraintGroup <rightBracket> ( KleeneOperator )? ( <colon> ( <ident> \| <integer> ) )? |
| ``` // ComplexPatternElement ``` |  |  |
| KleeneOperator | ::= | ( <kleeneOp> ) |
|  | \| | ( <leftSquare> ( <integer> ( <comma> <integer> )? ) <rightSquare> ) |
| ``` // KleeneOperator ``` |  |  |
| Constraint | ::= | ( <pling> )? <ident> ( ( FeatureAccessor <attrOp> AttrVal ) \| ( <metaPropOp> <ident> <attrOp> AttrVal ) \| ( <ident> ( ( <leftBrace> Constraint <rightBrace> ) \| ( Constraint ) ) ) )? |
| ``` // Constraint  //attribute values: strings, identifers (=strings), integers, floats, //booleans ``` |  |  |
| FeatureAccessor | ::= | ( <period> <ident> ) |
| ``` // attribute values: strings, identifers (=strings), integers, floats, //                   booleans ``` |  |  |
| AttrVal | ::= | ( ( <string> \| <ident> \| <integer> \| <floatingPoint> \| <bool> ) ) |
|  | \| | ( TemplateCall ) |
| TemplateCall | ::= | <leftSquare> <ident> ( <ident> <assign> AttrVal ( <comma> )? )\* <rightSquare> |
| RightHandSide | ::= | Action ( <comma> Action )\* |
| ``` // RightHandSide   // actions return 2 strings, one for the name of the block, and // one for the block itself. if the name is null, it is an anonymous block. // The checkLabel parameter indicates whether named blocks should check // at parse time that the label they refer to is bound.  Actions in // a MacroDef can't make this check at parse time, but instead the // check is done when the macro is referenced. ``` |  |  |
| Action | ::= | ( NamedJavaBlock \| AnonymousJavaBlock \| AssignmentExpression \| <ident> ) |
| ``` // Action   // A :bind { ... } code block.  The checkLabel parameter // indicates whether or not we should check *at parse time* that the // :bind label is valid.  Assignments that are the body of a MacroDef // can't check this at parse time but will be checked at reference time ``` |  |  |
| NamedJavaBlock | ::= | <colon> <ident> <leftBrace> ConsumeBlock |
| ``` // NamedJavaBlock ``` |  |  |
| AnonymousJavaBlock | ::= | <leftBrace> ConsumeBlock |
| ``` // AnonymousJavaBlock   // A :bind.Type = {features} assignment.  The checkLabel parameter // indicates whether or not we should check *at parse time* that the // :bind label is valid.  Assignments that are the body of a MacroDef // can't check this at parse time but will be checked at reference time ``` |  |  |
| AssignmentExpression | ::= | ( <colon> \| <colonplus> ) <ident> <period> <ident> <assign> <leftBrace> ( <ident> <assign> ( AttrVal \| ( <colon> <ident> ( ( <period> <ident> ( <period> \| <metaPropOp> ) <ident> ) \| ( <metaPropOp> <ident> ) ) ) ) ( <comma> )? )\* <rightBrace> |
| ``` // AssignmentExpression ``` |  |  |
| appendSpecials | ::= | *java code* |
| ``` // appendSpecials ``` |  |  |
| ConsumeBlock | ::= | *java code* |
